# Prevalence and bacterial etiology of subclinical mastitis in goats reared in organized farms

**DOI:** 10.14202/vetworld.2018.20-24

**Published:** 2018-01-13

**Authors:** A. K. Mishra, Nitika Sharma, D. D. Singh, K. Gururaj, Abhishek, Vijay Kumar, D. K. Sharma

**Affiliations:** 1 Division of Animal Health, ICAR-Central Institute for Research on Goats, Makhdoom, Farah, Mathura - 281 122, Uttar Pradesh, India; 2 Department of Veterinary Pathology, College of Veterinary Sciences, N.D.U.A.&T., Faizabad - 224 229, Uttar Pradesh, India; 3 Division of Bacteriology, ICAR-Indian Veterinary Research Institute, Izatnagar - 243 122, Uttar Pradesh, India

**Keywords:** California mastitis test, coagulase-negative *Staphylococci*, goat, somatic cell count, subclinical mastitis

## Abstract

**Aim:**

Assessment of the status of subclinical mastitis (SCM) in Jamunapari and Barbari goats in Indian organized farms, the involvement of bacterial pathogens and their sensitivity to antibiotics.

**Materials and Methods:**

A total of 181 composite milk samples were aseptically collected from the apparently healthy Barbari (n=95) and Jamunapari (n=86) goats. The California mastitis test (CMT) and somatic cell count (SCC) were used to diagnose SCM. The milk samples with CMT scores of 0 and +1 were considered as negative, while the samples with the score of +2 or +3 were taken as positive, and further, the positive samples were used for the bacteriological examination. An antibiotic sensitivity test was performed by disk diffusion method using seven commercially available antibiotic discs.

**Results:**

All the samples having CMT score of +2 or +3 demonstrated SCC more than 1 million. Overall, the prevalence of SCM in the goats was assessed as 19.89% (36/181). The prevalence of SCM in Barbari and Jamunapari goats was found as 24.21% (23/95) and 15.12% (13/86), respectively. Out of 11 isolates of Staphylococci, 9 isolates were identified as coagulase-negative Staphylococci (CNS), whereas 2 isolates were found as Staphylococcus aureus. The identified bacterial isolates (n=30) did not show antibiotic resistance.

**Conclusion:**

The current investigation showed the considerable prevalence of SCM among Jamunapari and Barbari goats which may have a negative impact on quantity and quality of the milk. CNS was found as the most prevalent cause of SCM in the goats. Negligible antibiotic resistance was found among the identified udder pathogens.

## Introduction

Mastitis, inflammation of mammary gland, is one of the most important diseases of dairy animals worldwide [1]. Economically, it is one of the most devastating diseases affecting the dairy animals not only in India but also throughout the world [2]. The economic losses are attributable to reduced milk production, discarded/poor quality milk, early culling, cost of veterinary services, decreased export of milk as well as milk products and the extra cost of management [3]. Mastitis affects both quantity and quality of milk and is characterized by physical, chemical, microbiological, and pathological changes in the udder and milk [4]. Mastitis in goats, such as cattle and buffaloes, is also an economically important disease worldwide [5]. Sub-clinical mastitis (SCM) is the most common in goats [6], is 15-40 times more prevalent than the clinical form [7]. In SCM, milk appears normal with no visible abnormalities in the mammary tissue of the affected goat. SCM usually precedes the clinical form and constitutes a reservoir of microorganisms which act as a source of infection to the healthy animals [3]. *Staphylococci* have been reported as the most common pathogen group associated with SCM in dairy goats [8,9]. *Staphylococcus aureus*, a coagulase-positive *Staphylococci*, has been found as the most common bacterial pathogen associated with clinical mastitis in dairy goats, whereas coagulase-negative *Staphylococci* (CNS) have been reported as the most prevalent in SCM in the goats [10]. *Streptococci* spp. are considered as the most common cause of the clinical as well as SCM in goats after *Staphylococci* [11,12].

Epithelial cells and leukocytes present in milk in response to intramammary infection are considered as milk somatic cells [13]. Milk somatic cells are good indicators of intramammary infection and can be employed to assess the level or occurrence of SCM in herds or individual animal [13]. In addition to somatic cell count (SCC), California mastitis test (CMT) is commonly used to detect SCM in animals [14].

The current study was conducted to assess the status of the SCM in Jamunapari and Barbari goats in organized farms, and the bacterial pathogens causing SCM and their susceptibility to the antibiotic.

## Materials and Methods

### Ethical approval

The approval from the Institutional Animal Ethics Committee to carry out the current study was not required as no invasive procedure on the animals was performed.

### Collection of samples

Milk samples were aseptically collected from both halves (composite milk) the apparently healthy goats of Barbari and Jamunapari breed under the organized farming system at ICAR-Central Institute for Research on Goats (ICAR-CIRG), Makhdoom, Mathura, Uttar Pradesh (India), as per the protocol recommended by National Mastitis Council [15]. Briefly, the teats were wiped with swabs soaked in 70% ethanol and thereafter, few streams of milk were discarded. Then, 10-15 ml of milk was collected into a sterile tube, labeled, and immediately brought to the Pathology Laboratory, Division of Animal Health, ICAR-CIRG, Makhdoom, Mathura, India. The samples were kept at 4°C and immediately checked for SCM, and then cultured within 24 h of their collection.

### Diagnosis of SCM

The CMT [16] and SCC method [17] were used to diagnose SCM. The CMT scores were graded as 0, +1, +2, and +3 according to the degree of reaction [18]. The milk samples with CMT scores of 0 and +1 were considered as negative, while the samples with score of +2 or +3 were taken as positive [16,18]. In short, CMT was performed using 3-4 ml of goat milk, to which an equal volume of CMT reagent was mixed immediately by swirling/circular motion. The reaction was graded by intensity of gel formation and color change. In this study, the goats having SCC ≥1 million per ml of milk were considered as positive for SCM [18]. Milk SCC was determined as suggested by Mishra *et al*. [4]. In summary, a uniform smear over the pre-drawn one square centimeter area was made on a clean glass slide using 10 μl of milk. The smear was stained with Newman’s Lampert stain (HiMedia, India). The counting of cells in 10 different fields was performed and was repeated thrice per smear to assess an average number of somatic cells in 30 fields. The total number of cells in the milk was assessed by multiplying a total number of cells in 10 fields to the working factor of the microscope, and was expressed per ml of milk sample.

### Bacteriological examination of milk samples

The milk samples having CMT score of +2 and +3 were taken for the bacteriological examination. The examination was done as per the method recommended by Mbindyo *et al*. [19]. Briefly, 10 microliters of milk were spread over 5% sheep blood agar plate by the quadrant streaking method, and the plate was incubated at 37°C for 24-48 h, and examined for characteristic bacterial colonies on the medium. Pure colonies on blood agar were further streaked on brain heart infusion agar, and incubated aerobically at 37°C for 24-48 h. At this level, the bacteria were tentatively identified on the basis of cultural, morphological and Gram’s reaction [20,21]. Further identification of bacteria was done using selective media such as Mannitol salt agar, Baird Parker medium, Edward medium, MacConkey agar, and Eosin methylene blue agar and by employing various biochemical tests such as catalase, oxidase, IMViC, coagulase, urease, nitrate, and sugar fermentation [22,23].

### Antibiotic sensitivity test

Antibiotic sensitivity test was performed by disk diffusion method on Mueller-Hinton medium (HiMedia, India) as per the procedure recommended by National Committee of Clinical Laboratory Standards [24] using 7 commercially available antibiotic discs (HiMedia, India), namely, amikacin, methicillin, amoxicillin, amoxicillin plus cloxacillin, cefoperazone, amoxicillin+clavulanic acid, and gentamicin. Briefly, the broth culture of bacterial isolate matching to 0.5 McFarland solutions was spread on Muller-Hinton agar thin smear. Zones of inhibition (in mm) produced by discs were noted after 16-18 h of incubation at 35-37°C. The zones were compared with the standards provided by the manufacturer to determine their susceptibility to the antibiotics.

### Statistical analysis

Data obtained from CMT test and SCC was used to determine the prevalence of the SCM in Barbari and Jamunapari goats. The effect of the variables such as age, parity, and breed on the prevalence of the disease was determined using Chi-square (*χ*^2^) test through Statistical Package for the Social Sciences.

## Results

In this study, the potential risk factors (age, parity, and breed) were taken into consideration to see the association between the incidence of the SCM in the lactating goats and the said factors. There was no statistically significant association found between the risk factors such as age (p=0.411), parity (p=0.12), and breed (p=0.28) and occurrence of the disease (Table-1). A total of 181 milk samples were collected, out of which, 95 were from Barbari goats and rest 86 were from Jamunapari goats. 23 milk samples from Jamunapari goats and 13 milk samples from Barbari goats showed CMT score of either +2 or +3, indicating the presence of SCM in the goats. All the samples having CMT score of +2 or +3 demonstrated SCC more than 1 million, but statistically, no significant difference was observed between the two by Chi-square analysis (p>0.05). Overall, the prevalence of SCM in the goats was assessed as 19.89% (36/181). The prevalence of SCM in Barbari and Jamunapari goats was found as 24.21% (23/95) and 15.12 % (13/86), respectively. In Barbari goats, the prevalence varied from 16.26% to 34.28% whereas in Jamunapari goats, it varied from 8.6% to 24.6% (Table-1), but statistically, no significant difference was observed between the two by Chi-square analysis (p>0.05). A total of 11 isolates of *Staphylococci* from 36 samples were isolated and identified on the basis of cultural, Gram’s reaction, morphological, and biochemical characteristics. Out of 11 isolates, 9 isolates were identified as CNS, whereas 2 isolates were determined as *S. aureus*. The details of the isolated and identified bacterial pathogens associated with SCM in the goats are given in Table-2. A total of 30 bacterial isolates comprising *Staphylococci* spp. (n=11), *Streptococci* spp. (n=7), *Bacilli* (n=8), *Escherichia coli* (n=2), *Mannheimia haemolytica* (n=1), and *Arcanobacterium pyogenes* (n=1) were evaluated for their antimicrobial susceptibility pattern against seven antibiotics. Antibiotic resistance was not observed among the bacterial isolates.

**Table-1:** Prevalence and association of risk factors with subclinical mastitis in goats.

Risk factor group	Number of goats examined	Goats with subclinical mastitis	Prevalence of subclinical mastitis at		χ^2^-value	p value

			Point prevalence	95% confidence level		
Age					0.677 (df=1)	0.411
<4 years	115	25	21.74	14.81-30.6		
>4 years	66	11	16.67	9.01-28.29		
Parity by number					4.11 (df=2)	0.12
1-3	32	9	28.13	14.4-46.98		
3-5	62	15	24.19	14.6-37.7		
>5	87	12	13.79	7.6-23.24		
Breed					2.34 (df=1)	0.12
Barbari	95	23	24.21	16.26-34.28		
Jamunapari	86	13	15.12	8.6-24.83		
Total	181	36	19.81	14.49-26.6		

NS=Not significant (p>0.05), df=Degrees of freedom

**Table-2:** Prevalence of the udder pathogens associated with subclinical mastitis in goats.

Pathogen	Number of isolates from Jamunapari	Number of isolates from Barabari	Prevalence (%)
Coagulasenegative *Staphylococci*	4/13	5/23	25.0%
*Bacillus* spp.	3/13	5/23	22.22%
*Streptococcus* spp.	2/13	5/23	19.44%
*Staphylococcus aureus*	1/13	1/23	5.55%
*Escherichia coli*	1/13	1/23	5.55%
*Mannheimia haemolytica*	0/13	1/23	2.77%
*Arcanobacterium pyogenes*	0/13	1/23	2.77%

## Discussion

Mastitis in goats is an important pathological condition, and is responsible for serious economic loss to goat farmers; and it is mainly encountered in goats in subclinical form [25]. SCM cannot be diagnosed by general physical examination such as palpation and organoleptic examination [25]. Overall, the prevalence of SCM in the goats was assessed as 19.89% (36/181). This finding is similar to that reported by Contreras *et al*. [26]. The prevalence of SCM in Barbari and Jamunapari goats was determined as 24.21% (23/95) and 15.12% (13/86), respectively. However, some false negative results can occurred due to the CMT classification used in our study. In the study, no statistically significant association between prevalence of the disease and risk factors such as age and parity was found. The finding is supported by the study conducted by Haftay *et al*. [27]. Statistically, no significant difference in the prevalence of the disease in Barbari and Jamunapari goats was found as determined by Chi-square analysis, probably due to the low samples number. However, the potential variability in the prevalence among the breeds may be attributed to the difference in genetic resistance, hygiene, milking practices, management systems, and technical knowledge of the investigators [28] along with methods used for the diagnosis of the disease. Study/investigation of the pathogens associated with intramammary infections helps in designing effective preventive and control strategies against mastitis such as bacterins and vaccines [29]. In our study, CNS were found to be the most prevalent bacteria in causing SCM in the goats which are supported by the earlier reports [16,25]. *Bacillus* spp. were found as the second most common cause of SCM in goats after the CNS which are in agreement with the study of Islam *et al*. [28]. The bacterial agents associated with SCM in goats were evaluated for their antimicrobial susceptibility pattern against seven commercially available antibiotics. Antibiotic resistance was not observed among the bacterial isolates. This finding is in accordance with the previous reports of Mbindyo *et al*. [19] and Brahma *et al*. [30] in which they did not find significant level of antibiotic resistance among the targeted isolates. Lack of the antibiotic resistance among the bacterial isolates may be attributed to proper and judicious use of antibiotics as per the standard recommendations [4] and non-use of antibiotics by farmers due to financial constraints.

## Conclusion

The current investigation showed the considerable prevalence of the SCM among Jamunapari and Barbari goats which may have negative impact on the health and production. Thus, there is need to improve managemental practices in the farms to decrease the prevalence of the disease to a possible lower limit. Most of the recommended antibiotics can be used against SCM at the farms because the antibiotic resistance among the identified udder pathogens was found negligible. Further, there is an urgent need to carry out a detailed epidemiological investigation to assess the actual prevalence of SCM at national level.

## Authors’ Contributions

AKM contributed in designing the experiment, sample-collection and isolation of the bacteria. NS did the prevalence study with respect to the SCM. DDS prepared the manuscript. KG contributed in the bacterial identification. Abhishek corrected the manuscript. VK did statistical analysis of data. DKS contributed in designing the experiment. All authors read and approved the final manuscript.
